# Advantage of a higher position of the tracheostoma with glottic closure for preventing complications related to tracheostomy tube: a retrospective cohort study

**DOI:** 10.1186/s12893-022-01505-2

**Published:** 2022-02-11

**Authors:** Yuji Kanazawa, Yasuhisa Kurata, Miki Nagai, Kenji Inoue, Fumihito Nozaki, Atsushi Mori, Mariko Ishihara, Mioko Mori, Tomohiro Kumada, Minoru Shibata, Takeo Kato, Masako Nakai, Makoto Kano

**Affiliations:** 1 Department of Otolaryngology, Shiga Medical Center for Children, 5-7-30 Moriyama, Moriyama, 524-0022 Japan; 2 Department of Diagnostic Imaging and Nuclear Medicine, Kyoto University Graduate School of Medicine, 54 Shogoinkawaharamachi, Sakyoku, Kyoto 606-8507 Japan; 3 Department of Otolaryngology, Sakai City Medical Center, 1-1-1, Ebarajicho, Nishiku, Sakai, 593-8304 Japan; 4 Department of Pediatrics, Shiga Medical Center for Children, 5-7-30 Moriyama, Moriyama, 524-0022 Japan; 5 Kumada Kids Family Clinic, 454-4 Kanegamorimachi, Moriyama, 524-0045 Japan; 6 Department of Otorhinolaryngology, Head and Neck, Ohara General Hospital, 6-1 Uwamachi, Fukushima, 960-8611 Japan

**Keywords:** Glottic closure, Laryngotracheal separation, Tracheo-brachiocephalic artery fistula, Computed tomography, Tracheostomy tube, Tracheo-innominate fistula

## Abstract

**Background:**

Surgery to prevent aspiration has complications related to tracheostomy tube, such as the trachea-brachiocephalic artery fistula. Glottic closure procedure makes tracheostoma at a position higher than the first ring of the trachea and theoretically has a potential to prevent such complications owing to a longer distance between the tip of tracheostomy tube and the tracheal membrane adjacent to the brachiocephalic artery. Our aim is to evaluate the safety of glottic closure in neurologically impaired patients by comparing outcomes with laryngotracheal separation.

**Methods:**

This study is a single-center retrospective study from 2004 to 2019, using data of 15 and 12 patients who underwent glottic closure (GC) and laryngotracheal separation (LTS). The primary outcome was the incidence of postoperative complications induced by tracheostomy tube placement and adjustment of the tracheostomy tube position to prevent these complications, such as by converting to a length-adjustable tube and/or placing gauze between the skin and tube flange. Additionally, we analyzed the anatomical relationship between the tracheostomy tube tip and brachiocephalic artery and measured the distance between them using postoperative CT images.

**Results:**

No patients in either group had trachea-brachiocephalic artery fistula. Erosion or granuloma formation occurred in 1 patient (7%) and 4 patients (33%) in the GC and LTS groups, respectively. Adjustment of the tracheostomy tube was needed in 2 patients (13%) and 6 patients (50%) in the GC and LTS groups. CT revealed a higher proportion of patients with the tracheostomy tube tip superior to the brachiocephalic artery in GC than LTS group. The mean tracheostoma-brachiocephalic artery distance was 40.8 and 32.4 mm in the GC and LTS groups.

**Conclusions:**

Glottic closure reduces the risk of postoperative complications related to a tracheostomy tube. This may be due to the higher position of the tracheostoma at the level of the cricoid cartilage, increasing the distance between the tracheostoma and brachiocephalic artery.

**Supplementary Information:**

The online version contains supplementary material available at 10.1186/s12893-022-01505-2.

## Background

**Fig. 1:**
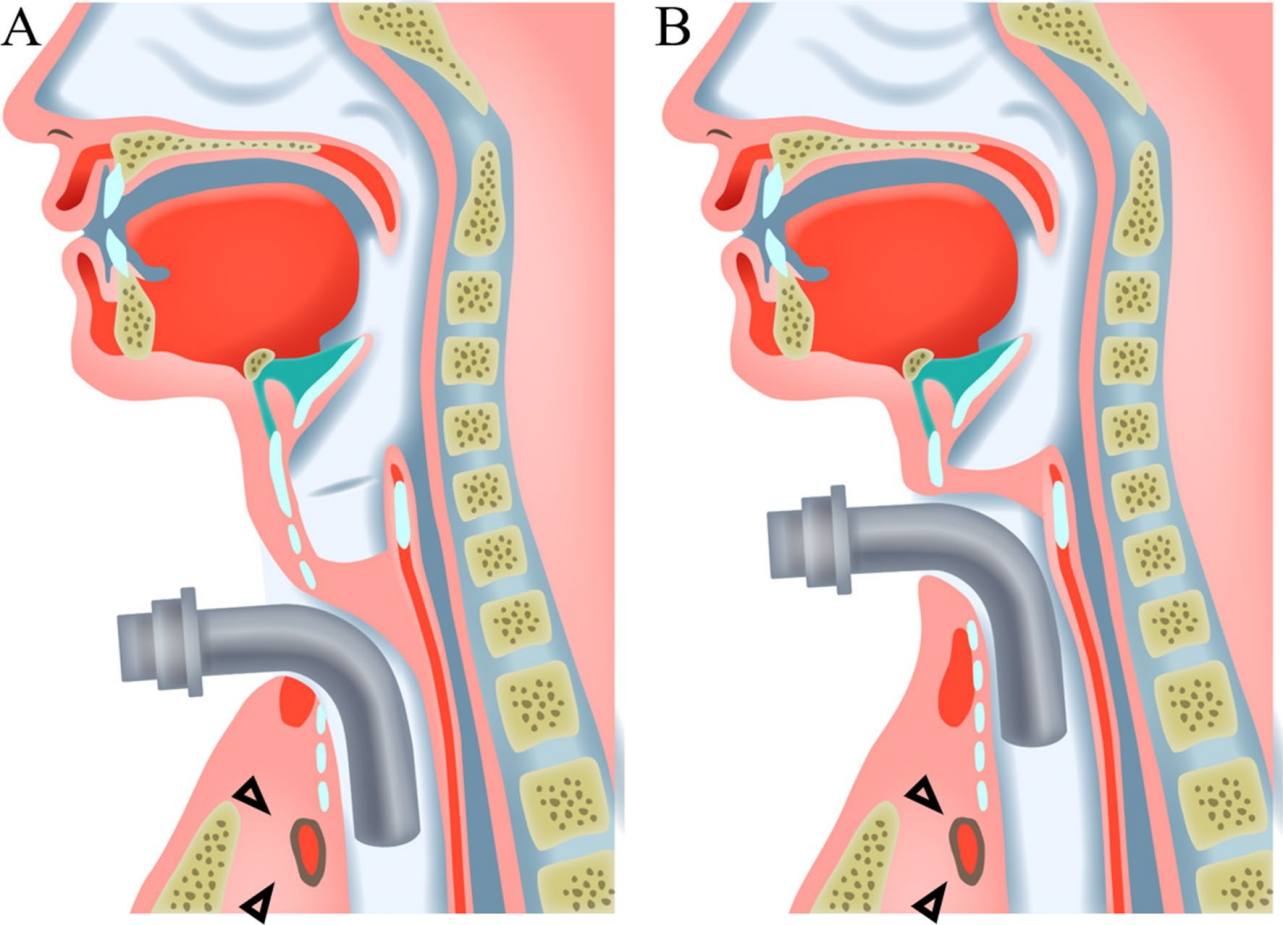
Anatomical relationship between the tracheostomy tube and brachiocephalic artery after laryngotracheal separation (**A**) and glottic closure (**B**). **A** A lower position of the tracheostoma can increase the risk of trachea- brachiocephalic fistula due to contact with the tip or cuff of the tracheostomy tube. **B** Increasing the distance between the tracheostoma and brachiocephalic artery by adopting a higher position can prevent this contact, possibly reducing the risk of trachea- brachiocephalic fistula. The arrowhead indicates the brachiocephalic artery

Among various surgical techniques to prevent aspiration due to dysphagia, laryngotracheal separation (LTS) is commonly performed for neurologically impaired children [1, 2]. This procedure creates a tracheostoma at the level of the trachea and often results in the tracheostomy tube making contact with the anterior part of the tracheal membrane near the brachiocephalic artery (Fig. 1A), thereby increasing the risk of trachea-brachiocephalic artery fistula (TBF), a life-threatening complication [3–6]. This risk is particularly high in children in whom the trachea has shifted to the sternum due to a thoracic deformity or scoliosis, so careful management of the tracheal tube is needed [3].

Recently, Kano devised a new surgical technique to prevent aspiration, named glottis closure procedure (GC) [7]. In this procedure, the anterior part of cricoid and thyroid cartilage are exposed and removed, the cricothyroid membrane is incised, and the cricoid and arytenoid cartilage are separated. Tracheostoma is made at a position higher than the first ring of the trachea in GC. This higher positioning results in a longer distance between the artery and tracheal tube than with LTS (Fig. 1B) and thus reduces the risk of TBF [3]. Based on this concept, our facility decided to adopt the GC procedure devised by Kano instead of LTS from 2017. However, the clinical benefits of this higher level of tracheostoma remain unclear. In particular, whether or not GC reduces the incidence of postoperative complications related to the tracheostomy tube, such as TIF, in neurologically impaired patients with or without scoliosis has yet to be determined.

We herein report the efficacy of GC, which creates a tracheostoma at a higher position than conventional LTS. We reviewed the clinical data of neurologically impaired patients who underwent GC or LTS, focusing on postoperative complications, including TBF, erosion and granuloma formation, and ways to prevent these issues. In addition, we measured the distance from the tracheostoma to the anterior part of the trachea adjacent to the brachiocephalic artery using cervical computed tomography (CT) images in order to assess the anatomical characteristics of tracheostoma with GC compared to that with LTC.

## Methods

### Patients

This study is a single-center retrospective study from 2004 to 2019. In this period, 27 neurologically impaired patients underwent aspiration prevention surgery. Among them, GC was performed for 15 patients from 2017 to 2019 (GC group), and LTS was performed for 12 neurologically impaired patients from 2003 to 2014 (LTS group). The basic characteristics and clinical course are summarized in Additional file 1. Tracheostomy before surgery had been performed in 2 patients (13%) and 4 patients (33%) in the GC and LTS group, respectively. The mean age at the operation was 7 years old (standard deviation [SD] = 10.2 years old) and 9.7 years old (SD = 7.3 years old) in the GC and LTS groups, respectively. The follow-up period was 2.1 years (range, 1–3 years) and 11.6 years (range, 6–17 years) in the GC and LTS groups, respectively. No operative or early postoperative complications occurred, including anastomotic leakage, and the rate of pneumonia-related hospitalization per year decreased after surgery in both groups. Overall, a respirator was needed all day in 8 patients (30%) and only at night in 15 patients (56%). Scoliosis was diagnosed by one radiologist and export of spine surgery based on the Cobb method, measuring the angle formed by a line drawn along the upper and lower endplate of the upper and lower ends of the cervical vertebrae using spine posterior-anterior radiographs [8]. Cobb angle was 33.9 ± 21.3, 20.2 ± 15.2 degrees in the GC and LTS groups. Scoliosis in the cervical region was defined by a Cobb angle greater than 20 degrees and present in 7 patients (47%) and 1 patient (8%) in the GC and LTS groups, respectively. The proportion of patients with cervical scoliosis was higher in the GC group than in the LTS group. Tracheostomy had been previously performed in 2 (13%) and 4 patients (33%) in the GC and LTS groups, respectively. A canula-free state was only possible in 4 patients (27%) in the GC group; some patients wanted to avoid this state because of the nightly need for tracheostomy tube insertion for respiration. The study’s protocol was approved by the ethics committee of Shiga Medical Center for Children. The requirement for informed consent was not applicable because this study was performed using deidentified data obtained from patient medical records.

### Postoperative complications related to the tracheostomy tube

We defined postoperative complications as the incidence of TBF, granuloma formation, and extra management for tracheostomy tube to prevent these problems. In our department, tracheas with and without tracheostomy tubes were regularly checked via bronchoscopy after surgery. The tracheostomy tube type was changed to adjust the length and angle in cases where the tracheostomy tube tip made contact with the tracheal wall or granuloma formation. When it was difficult to prevent such contact merely by changing the canula type, management was attempted by making the distance using a few stripes of gauze placed between the skin and tube flange and a length-adjustable tube [5]. If this approach did not work, a custom-made tube was considered. We examined the incidence of these postoperative complications, including granuloma formation and contact between the tracheostomy tube tip and the trachea eligible for use of gauze and a special type of tracheostomy tube, during one year after surgery. These results were then compared between the GC and LTS groups. In addition, an analysis stratified by the presence of scoliosis in the cervical region was performed, because this feature has been reported to cause deformity of the trachea and thereby increase the risk of postoperative complications, such as TBF [3].

### The acquisition and evaluation of CT findings

**Fig. 2:**
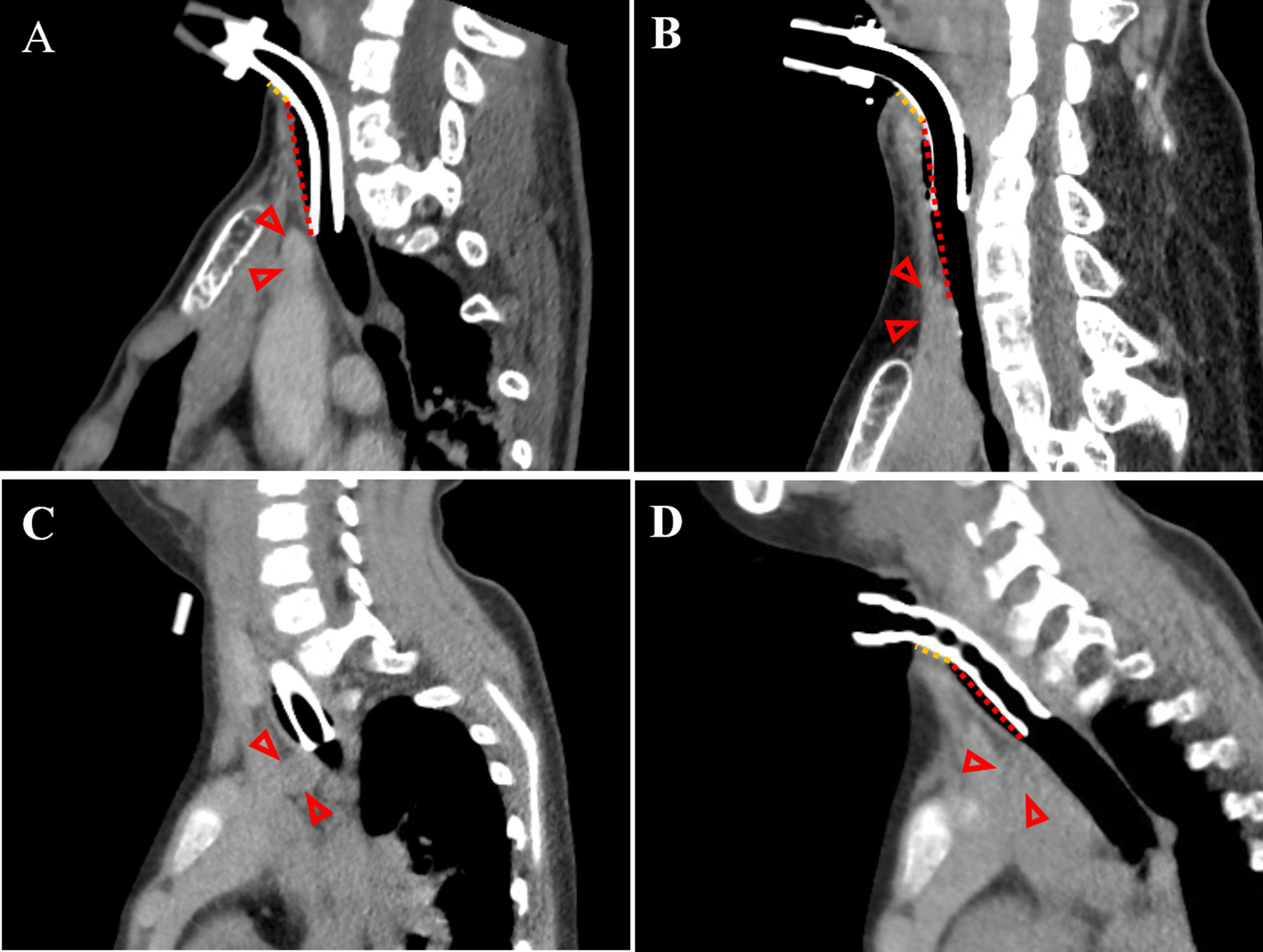
Measurement of the distance between the tracheostoma and brachiocephalic artery (TB length) using straight lines; the orange dot line passed from the tracheostoma to the anterior part of the trachea, while the red dot line passed from the trachea along the tracheal tube. A representative case is shown in **A** (case 2 in the LTS group) and **B** (Case 21 in the GC group). When the tracheostomy tube tract was not traced in one sagittal image because of deformity of the trachea in a patient with severe scoliosis (**C**, case 15 in the GC group), the axial image was reconstructed to measure the TB length in one image (**D**). The arrowhead indicates the brachiocephalic artery

Among 21 patients whose postoperative neck CT findings were available (12 in the GC group and 9 in the LTS group), the distance from the brachiocephalic artery to the tracheostoma was analyzed. An 80-slice and 4-slice multi-detector CT (MDCT) scanner (Toshiba Medical Systems, Tokyo, Japan) was used in the GC and LTS groups, respectively. The slice thickness was 3 mm and 5 mm in the axial and sagittal planes, respectively. In each patient, we selected a sagittal CT slice in which the location of both the tracheostoma and the brachiocephalic artery were able to be identified. We measured the distance from the inferior part of the tracheostoma and the anterior wall of the trachea adjacent to the brachiocephalic artery (TB length), because the anterior tracheal wall was found to be the most common site of erosion in previous studies [4, 5]. For this measurement of CT, two straight lines were used to increase the objectivity of the measurement; one line passed from the tracheostoma to the anterior part of the trachea, while the other line passed from the trachea along the tracheal tube (Fig. 2A, B). The TB length was determined by combining the length of these two lines as measured by the Image J software program. In cases of severe scoliosis in the cervical region, the axial CT plane was reconstructed to identify these two points in the one slice, using an image analysis system (SYNAPSE VINCENT; Fujifilm Corp., Tokyo, Japan) (Fig. 2C, D). This image processing was eligible for 4 and 3 patients in the GC and LTS groups, respectively. In addition, the relationship of the tip or cuff of the tracheostomy tube with the tracheal membrane adjacent to the brachiocephalic artery was evaluated as follows: tube tip is superior to the brachiocephalic artery, adjacent to the brachiocephalic artery, or inferior to the brachiocephalic artery [4, 5]. An endotracheal tube cuff pressure had been adjusted below 25 mm Hg to prevent tracheal ischemic complications [9, 10]. All measurements were performed by one otolaryngologist and one radiologist who were blinded to the patient information. Student’s *t*-test was used to examine the difference in the TB length between the GC and LTS group. Statistical significance was considered present at p < 0.05.

## Results

### Postoperative complications related to the tracheostomy tube

**Fig. 3:**
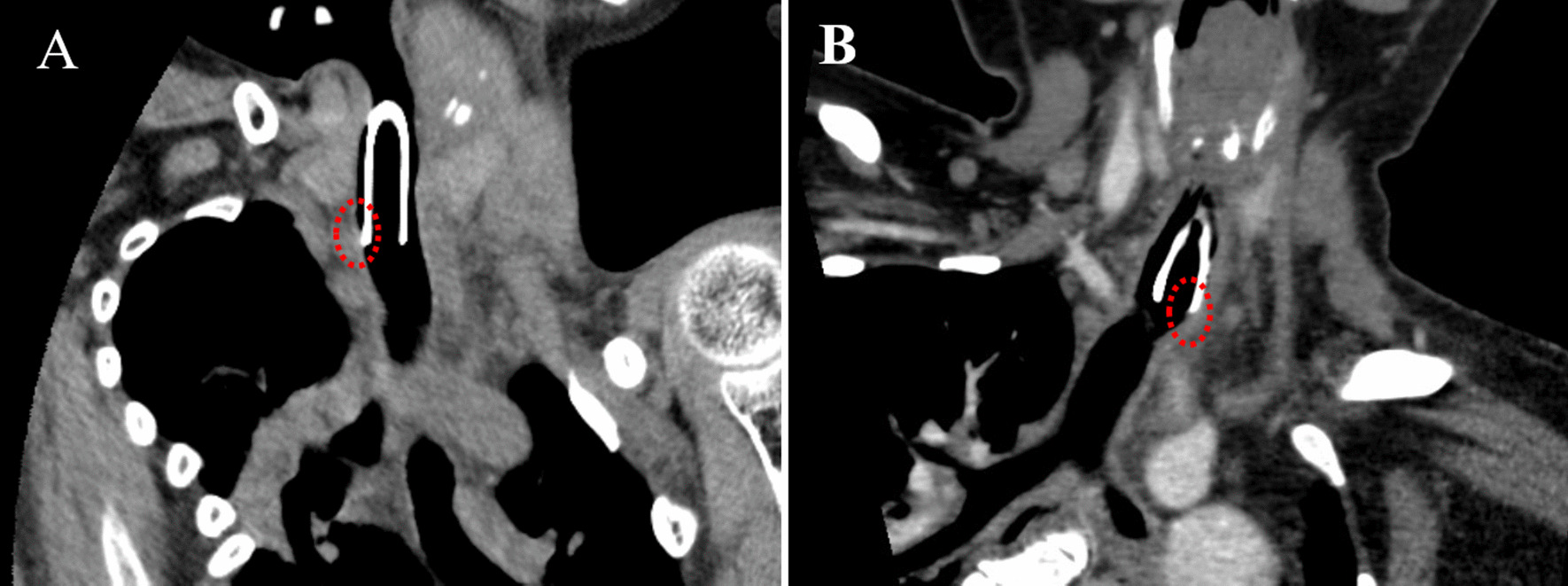
Coronal view of CT showing that the tip of the tracheostomy tube made contact with the trachea due to deformity of the trachea in cases of severe scoliosis (**A**: case 23, **B**: case 26). In these cases, the tracheal tube needed shortening

The postoperative complications of each patient are shown in Table 1. No patients had TBF, and there were no cases eligible for surgical management, including brachiocephalic artery dissection and manubrium resection. Granuloma formation in the anterior part of the trachea occurred in 1 (7%) and 4 patients (33%) in the GC and LTS groups, respectively, within 1 year after surgery. Management to prevent contact between the tracheostomy tube and the trachea was needed in 4 (27%) and 7 patients (58%) in the GC and LTS groups, respectively (Table 1). Among them, management with a few strips of gauze was needed in 3 (20%) and 6 patients (50%) in the GC and LTS groups, respectively, and converting the tracheostomy tube to a length-adjustable type or custom-made tube was needed in 2 (13%) and 2 patients (17%) in the GC and LTS groups, respectively. One patient in the LTS group needed a custom-made tracheostomy tube to adjust the angle of the tube because of repetitive granuloma formation of the trachea despite angle adjustment with gauze. Regardless of the presence or absence of scoliosis in the cervical region, the proportion of patients with these complications was lower in the GC group than in the LTS group (Table 2). Among patients with cervical scoliosis, three required shortening the tube to place it above the narrow tracheal space due to severe deformity of the trachea (Fig. 3).

**Table 1:** Characteristics and analysis results for each patient

Case No.	Ope– type	Ope– Age (years)	Sex	Scoliosis	Postoperative complications		Postoperative CT	
					Granuloma formation	Extra Management related to tracheostomy tube	TB length (mm)	Relation of tube tip to brachiocephalic. A
1	LTS	1	F	–	–	2 gauze	38	A
2	LTS	3	M	–	–	–	42	A
3	LTS	3	M	–	–	–	–	X
4	LTS	4	F	–	–	3 gauze	26	A
5	LTS	7	M	–	–	–	25	I
6	LTS	7	M	–	–	2 gauze	46	S
7	LTS	7	M	–	+	–	–	A
8	LTS	11	M	+	+	2 gauze/ Custom–made	30	I
9	LTS	14	F	–	–	–	25	S
10	LTS	17	F	–	+	3 gauze	25	A
11	LTS	17	F	–	+	3 gauze	–	X
12	LTS	25	F	–	–	Length–adjustable	35	S
13	GC	2	M	–	–	–	26	I
14	GC	2	M	–	–	–	–	X
15	GC	5	F	+	–	–	36	A
16	GC	10	F	–	–	–	–	X
17	GC	11	F	–	–	–	33	A
18	GC	11	M	–	–	–	41	A
19	GC	14	F	+	–	2 gauze	22	A
20	GC	14	M	–	–	–	37	A
21	GC	16	F	+	–	–	54	S
22	GC	20	F	+	–	–	46	S
23	GC	20	M	+	–	Length–adjustable	36	S
24	GC	26	M	+	–	–	–	X
25	GC	30	F	+	–	–	50	S
26	GC	31	F	+	+	2 gauze/ Length–adjustable	44	A
27	GC	33	F	+	–	2 gauze	65	S

*LTS* laryngotracheal separation, *GC* glottic closure, *S* Tracheostomy tube tip is superior to the brachiocephalic artery, *A* Tracheostomy tube tip is adjacent to the brachiocephalic artery, *I* Tracheostomy tube tip is inferior to the brachiocephalic artery, *X* CT data are missing

**Table 2:** Postoperative complications related to the tracheotomy tube in the two groups, stratified by the scoliosis status

	N	Postoperative complications
With scoliosis		
GC	7	3 (43%)
LTS	1	1 (100%)
Without scoliosis		
GC	8	2 (25%)
LTS	11	6 (55%)

*LTS* laryngotracheal separation, *GC* glottic closure

### The postoperative CT evaluation of the trachea

**Fig. 4:**
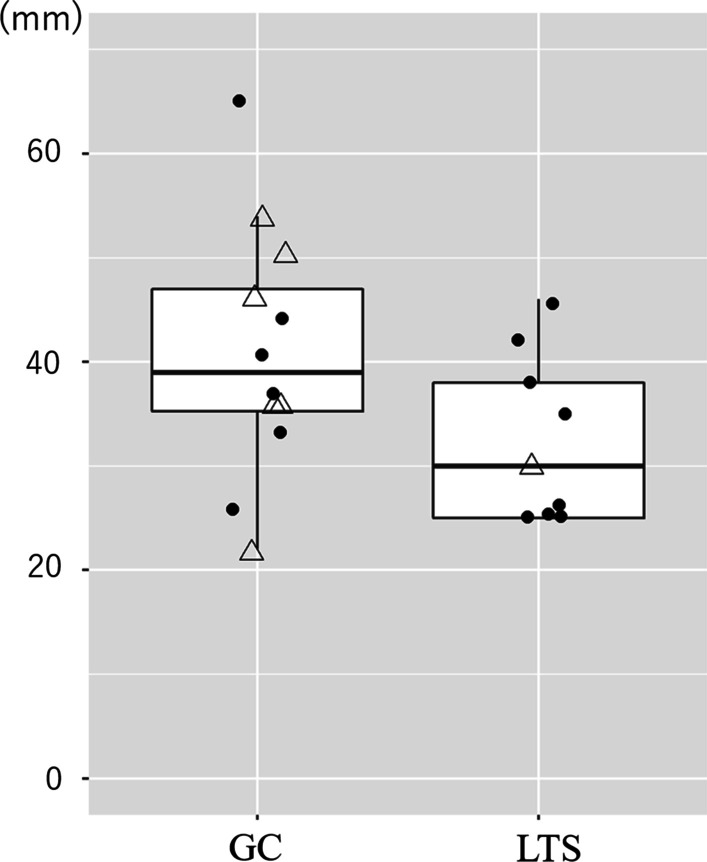
The distance from the tracheostoma to the brachiocephalic artery (TB length). The mean TB length was longer in the glottic closure group (GC) than in the laryngotracheal separation group (LTS). Patients with scoliosis of the cervical region are indicated with triangles. These data do not include the data of six patients whose CT images were not available

The TB length of each patient is shown in Table 1; Fig. 4. The mean TB length was 40.8 mm in the GC group and 32.4 mm in the LTS group. There was no significant difference between the two groups; the mean difference was 8.4 mm (95% confidence interval: − 1.3 to 18.1 mm, p = 0.08). We then evaluated the relationship of the tracheostomy tube tip to the brachiocephalic artery (Table 1). The tip was superior to the brachiocephalic artery in 6 (50%) and 3 patients (30%) in the GC and LTS groups, respectively.

## Discussion

This study investigated and compared the incidence of postoperative complications related to a tracheostomy tube between the GC and LTS procedures. The proportion of patients with postoperative complications, including those requiring extra management, was lower in the GC group than in the LTS group, suggesting that GC has a clinical advantage of safe management for preventing TBF. This advantage may be due to the creation of a longer distance between the tracheostoma and brachiocephalic artery with GC.

No patients in the present study had TBF, which is consistent with the rare incidence of TBF previously reported (<1% in patients with a tracheostomy tube) [11]. The low incidence of TBF may be due to the careful care and management related to the tracheostomy tube in each facility. Namely, it is important to monitor the anatomical relationship between the tip or cuff of the tracheostomy tube and the brachiocephalic artery and ensure that the tracheostomy tube fits the shape of the trachea [3, 5, 11]. In our facility, we employ two main strategies for adjusting the tracheostomy tube length: using a length-adjustable tube and placing gauze between the skin and tube flange. A length-adjustable tube is useful but expensive, whereas gauze can be used for minor adjustments of the tube position at little cost. Indeed, gauze was used in 33% of patients in this study, suggesting that the placement of gauze between the skin and tube flange is a useful option for adjusting the tracheostomy tube position.

The proportion of patients with postoperative complications, including granuloma formation of the tracheal wall and the need for gauze or a length-adjustable or custom-made tracheostomy tube, was lower in the GC group than in the LTS group. This is mainly because the tracheostomy tube was placed superior to the brachiocephalic artery in the GC group, as demonstrated by our CT analysis. This interpretation is in good accord with the postoperative advantage of a higher position of tracheostoma demonstrated previously [7]. In fact, our CT analysis showed a longer mean TB length in the GC group than in the LTC group, although this difference between the two groups was not significant. One possible explanation for no significance is that other factors associated with the TB length were not included in our analysis due to the small sample size, such as the age at operation, body height, and weight, all of which can affect the basic length of the trachea. Although further studies are needed to determine the anatomical advantages of GC using CT measurements, such as the TB length, the higher tracheostoma position with GC can help reduce the risk of postoperative complications related to the tracheostomy tube.

Another advantage of GC is that a higher tracheostoma position can prevent contact between the tracheal tube tip and the tracheal wall, even in patients with severe deformity of the trachea due to scoliosis. Anatomically, the trachea is more deformed at lower levels in patients with scoliosis, facilitating contact of the tip with the tracheal wall and increasing the risk of TBF [3, 5, 12]. Since the cervical and thoracic spine are common sites of scoliosis [13, 14], the tip of the tracheostomy tube can easily make contact with the tracheal wall in patients with scoliosis. In such patients, using a length-adjustable or custom-made tube should be considered. However, our analysis showed that some patients in the GC group did not require conversion of the tracheostomy tube type, suggesting that the higher tracheostoma position with GC is an anatomical advantage that helps prevent problems related to the tracheostomy tube in patients with scoliosis.

Importantly, however, we also found that the tracheostomy tube tip was actually adjacent to the brachiocephalic artery in half of the patients in the GC group. This is probably due to natural variations in the location of the brachiocephalic artery [4]. Postoperative complications related to the tracheostomy tube may occur depending on this variation, regardless of the operative procedure type. Further studies are therefore needed to clarify the efficacy of GC while taking into account variations in the position of the brachiocephalic artery in each patient using three-dimensional CT images.

## Conclusions

This study evaluated the efficacy of the GC procedure with regard to preventing postoperative complications, including tracheal tube management, in neurologically impaired patients. We found a low proportion of patients with postoperative complications, including those requiring extra management of the tracheostomy tube to prevent TBF, and a longer distance between the tracheostoma and the brachiocephalic artery in the GC group than in the LTS group. These findings suggested that GC can contribute to the safe management of the tracheostomy tube and allow us to easily adjust the length of the tube even in patients with tracheal deformity due to scoliosis. Further studies are warranted to evaluate this advantage of the GC using multicenter cohort.

## Supplementary Information

**Additional file 1.** Basic characteristic and clinical course in each patient, including primary disease, oral intake, prior-tracheostomy, pneumonia eligible for hospitalization, and the use of respirator and tracheostomy tube.

## Data Availability

The datasets used and/or analysed during the current study are available from the corresponding author on reasonable request.

## Abbreviations

GC: Glottic closure
LTS: Laryngotracheal separation
TBF: Trachea-brachiocephalic artery fistula
TB length: The distance from the inferior part of the tracheostoma and the anterior wall of the trachea adjacent to the brachiocephalic artery
