# Supplementary material for: Advantage of a higher position of the tracheostoma with glottic closure for preventing complications related to tracheostomy tube: a retrospective cohort study
Source: BMC Surg. 2022 Feb 11;22:50. doi: 10.1186/s12893-022-01505-2 (PMC8832853; doi:10.1186/s12893-022-01505-2)
Supplement: Supplementary file 1 — Additional file 1. Basic characteristic and clinical course in each patient, including primary disease, oral intake, prior-tracheostomy, pneumonia eligible for hospitalization, and the use of respirator and tracheostomy tube. [file 12893_2022_1505_MOESM1_ESM.pdf]

Advantage of a higher position of the tracheostoma with glottic closure for preventing complications related to tracheostomy tube: a retrospective cohort study

Case No	Ope-type	Ope-Age (y)	Sex	Primary disease"	Prior-tracheostomy	FOIS score		Pneumonia-hospitalization (day/year)		Respir-ator	Canula -free
						preop	postop	preop	postop		
1	LTS	1	F	Gaucher disease	-	1	1	0	19	day	-
2	LTS	3	M	WEST syndrome	+	1	1	30	0	night	-
3	LTS	3	M	White matter dystrophy	-	1	1	89	57	night	-
4	LTS	4	F	Cerebral palsy	-	1	1	96	16	-	-
5	LTS	7	M	Trisomy 18	+	1	1	99	0	day	-
6	LTS	7	M	Degenerative disease	-	1	1	108	0	night	-
7	LTS	7	M	Cerebral palsy	+	1	1	73	42	night	-
8	LTS	11	M	Degenerative disease	-	1	1	15	0	night	-
9	LTS	14	F	Congenital anomaly	+	1	1	15	0	-	-
10	LTS	17	F	Amyotrophic lateral sclerosis	-	1	3	39	9	-	-
11	LTS	17	F	Anoxic injury	-	1	1	246	0	-	-

12	LTS	25	F	Anoxic injury	-	1	1	69	0	day	-
13	GC	2	M	Brain stem bleeding	+	1	3	116	13	day	-
14	GC	2	M	Cerebral palsy	-	1	2	18	25	night	-
15	GC	5	F	West syndrome	-	1	1	29	45	night	-
16	GC	10	F	West syndrome	-	1	2	25	86	night	-
17	GC	11	F	Anoxic injury	-	2	3	0	0	night	+
18	GC	11	M	Pelizaeus-Merzbacher disease	-	1	2	44	0	night	+
19	GC	14	F	Anoxic injury	+	1	1	3	0	night	+
20	GC	14	M	Cerebral palsy	-	1	1	36	14	night	-
21	GC	16	F	Fukuyama muscular dystrophy	-	1	2	36	15	day	-
22	GC	20	F	Cerebral palsy	-	3	3	39	32	night	-
23	GC	20	M	Cerebral palsy	-	1	1	13	0	night	+
24	GC	26	M	Mitochondrial disease	-	1	1	25	9	day	-
25	GC	30	F	Cerebral palsy	-	3	2	70	0	night	-
26	GC	31	F	Anoxic injury	-	1	1	19	0	day	-
27	GC	33	F	Rett syndrome	-	1	1	75	0	day	-

FOIS: Functional Oral Intake Scale[1]

1. Crary MA, Mann GD, Groher ME. Initial psychometric assessment of a functional oral intake scale for dysphagia in stroke patients. Arch Phys Med Rehabil 2005; 86:1516-1520.
